# FZL, a dynamin-like protein localized to curved grana edges, is required for efficient photosynthetic electron transfer in *Arabidopsis*

**DOI:** 10.3389/fpls.2023.1279699

**Published:** 2023-09-28

**Authors:** Yu Ogawa, Megumi Iwano, Toshiharu Shikanai, Wataru Sakamoto

**Affiliations:** ^1^ Institute of Plant Science and Resources, Okayama University, Kurashiki, Japan; ^2^ Graduate School of Biostudies, Kyoto University, Kyoto, Japan; ^3^ Department of Botany, Graduate School of Science, Kyoto University, Kyoto, Japan

**Keywords:** FUZZY ONION LIKE (FZL), *Arabidopsis*, chloroplast, thylakoid, thylakoid structure, photosynthetic electron transfer

## Abstract

Photosynthetic electron transfer and its regulation processes take place on thylakoid membranes, and the thylakoid of vascular plants exhibits particularly intricate structure consisting of stacked grana and flat stroma lamellae. It is known that several membrane remodeling proteins contribute to maintain the thylakoid structure, and one putative example is FUZZY ONION LIKE (FZL). In this study, we re-evaluated the controversial function of FZL in thylakoid membrane remodeling and in photosynthesis. We investigated the sub-membrane localization of FZL and found that it is enriched on curved grana edges of thylakoid membranes, consistent with the previously proposed model that FZL mediates fusion of grana and stroma lamellae at the interfaces. The mature *fzl* thylakoid morphology characterized with the staggered and less connected grana seems to agree with this model as well. In the photosynthetic analysis, the *fzl* knockout mutants in *Arabidopsis* displayed reduced electron flow, likely resulting in higher oxidative levels of Photosystem I (PSI) and smaller proton motive force (pmf). However, nonphotochemical quenching (NPQ) of chlorophyll fluorescence was excessively enhanced considering the pmf levels in *fzl*, and we found that introducing *kea3-1* mutation, lowering pH in thylakoid lumen, synergistically reinforced the photosynthetic disorder in the *fzl* mutant background. We also showed that state transitions normally occurred in *fzl*, and that they were not involved in the photosynthetic disorders in *fzl*. We discuss the possible mechanisms by which the altered thylakoid morphology in *fzl* leads to the photosynthetic modifications.

## Introduction

The light reactions of photosynthesis convert light energy into chemical energy, which all life on earth directly or indirectly depends on. Driven by the light energy absorbed by light harvesting complex (LHC) II and LHCI, water-derived electrons are transferred to ferredoxin, through thylakoid-embedded photosynthetic machineries such as photosystem (PS) II, cytochrome *b*_6_ *f* (Cyt *b*_6_ *f*) and PSI, and ultimately to NADP^+^, producing NADPH (linear electron flow/LEF). The transfer of electrons between each complex is mediated by the mobile electron carriers plastoquinone (PQ) and plastocyanin (PC). The electron transfer is coupled with proton transport into thylakoid lumen, developing an electrochemical gradient of protons, called proton motive force (pmf). Pmf is a sum of an electric field (ΔΨ) and a difference in proton concentration (ΔpH) and is used to produce ATP by CF_o_-CF_1_ ATP synthase (ATP synthase/ATPase).

To optimize the photosynthetic reactions under ever-changing light conditions in nature, plants have several regulatory mechanisms to swiftly adjust electron transfer in a time scale of seconds to minutes. The short-term mechanisms include nonphotochemical quenching (NPQ) of chlorophyll fluorescence, photosynthetic control, and state transitions. Recently, grana stacking dynamics is also counted as another regulatory strategy in some reports (Hepworth et al., 2021; Flannery et al., 2023). Both NPQ and photosynthetic control are induced by the ΔpH component of pmf (Cruz et al., 2005). The major component of NPQ, qE, is a mechanism to dissipate excess energy from light as heat, and is triggered by protonation of violaxanthin deepoxidase (VDE) and PsbS when thylakoid lumen is acidified (Li et al., 2000; Li et al., 2002). Photosynthetic control restricts the activity of Cyt *b*_6_ *f* in response to low luminal pH, slowing down the rate of electron transfer toward PSI (Stiehl and Witt, 1969; Munekage et al., 2001; Yamamoto and Shikanai, 2019). State transitions ensure efficient electron transfer by balancing the excitation pressure between PSII and PSI (Allen, 2003). When LHCII is dephosphorylated, it preferentially excites PSII (state 1). Meanwhile, when STN7 kinase is activated by reduced PQ pool and LHCII is phosphorylated, larger proportion of LHCII become energetically coupled with PSI (state 2) (Bellafiore et al., 2005; Tikkanen et al., 2008). Although state transitions were often associated with changes in grana stacking (see below), they have been demonstrated to be independent processes both of which are induced by STN7-dependent LHCII phosphorylation (Wood et al., 2019).

The thylakoid membrane of vascular plants, which harbors all the photochemical reactions and regulatory mechanisms described above, is particularly remarkable for its intricate architecture; cylindrical grana appressions are interlinked with each other via non-appressed stroma lamellae helically winding around them (Bussi et al., 2019; Rantala et al., 2020). This non-uniform structure is accompanied by heterogeneous distribution of photosynthetic machineries; PSII and LHCII are enriched on grana, PSI-LHCI and ATP synthase are located on stroma lamellae, and Cyt *b*_6_ *f* is likely distributed on all thylakoid domains (Rantala et al., 2020). Some PSIIs and PSIs coexist in grana-stroma lamellae interfaces denoted as grana margins, where state transitions mainly occur (Tikkanen et al., 2008; Rantala et al., 2020). To some extent, these major photosynthetic protein complexes determine the thylakoid structure. Grana appression is considered to be created by electrostatic attraction between charged stromal loops of LHCII proteins on opposite membranes (Barber, 1980), and the grana diameters become smaller when the electrostatic forces are modified by STN7-dependent LHCII phosphorylation (Pribil et al., 2014). In addition, looking at the fragmentary stroma lamellae observed in mutants specifically deficient in PSI, it is tempting to speculate that PSI complexes may contribute to expand stroma lamellae (Amann et al., 2004; Pribil et al., 2014). However, the unique thin and wide grana in *curt1* mutants pointed out that plants still need other specific proteins to maintain the thylakoid structure (Armbruster et al., 2013). CURVATURE THYLAKOID1 (CURT1) maintains the highly stacked grana structures by creating their bent edges with its membrane-curving ability. It has been reported that in *Arabidopsis curt1* mutants, PC-mediated electron transfer and state transitions are retarded (Pribil et al., 2018; Höhner et al., 2020), suggesting the significance of thylakoid shaping by membrane remodeling proteins in achieving efficient photosynthetic electron transfer and its fine-tuned regulation.

There is another membrane remodeling protein which presumably contributes to maintain the thylakoid structure: FUZZY ONION LIKE (FZL) (Gao et al., 2006). FZL is a dynamin-like protein, and it has been demonstrated *in vivo* to mediate fusion of thylakoid membranes (Findinier et al., 2019). In *Arabidopsis fzl* mutants, chloroplast size is larger and thylakoid morphology is disorganized (Gao et al., 2006). The observation of fragmented thylakoids in developing *fzl* chloroplasts and the immunolabeling of FZL-GFP at grana-stroma lamellae interfaces suggested that FZL fuses grana and stroma lamellae at grana peripheries during thylakoid biogenesis (Liang et al., 2018). However, there has been a disagreement about the sub-chloroplast localization of FZL; Gao et al. (2006) showed the dual localization of FZL to thylakoids and envelopes, whereas Patil et al. (2018) concluded it was primarily localized to envelopes. In addition, the full development of thylakoid membranes in *fzl* knockout mutants implies that FZL is dispensable in thylakoid biogenesis (Gao et al., 2006). Therefore, the function of FZL remains to be determined both in terms of membrane remodeling and physiology. Patil et al. (2018) reported the altered photosynthetic performance of *fzl* mutant, but the plant samples used for their photosynthetic analysis may have suffered from indirect damages due to the differences in growth conditions, which makes it harder to determine the direct effects of *fzl* mutations; the analysis was focused on the midvein regions of leaves of plants grown under LED lights of 200 μmol photons m^-2^ sec^-1^ because the pale green phenotype was manifest in those leaf parts. Therefore, in this study, we reevaluated the sub-chloroplast localization of FZL and reexamined the photosynthetic phenotype of *Arabidopsis fzl* mutants using the whole leaves of plants grown under milder conditions. In agreement with Liang et al. (2018), we detected FZL at edges of grana. Our photosynthetic analysis suggests that the thylakoid membrane remodeling by FZL is required to promote electron transfer and properly induce NPQ.

## Results

### FZL is enriched on curved edges of grana

**Figure 1:**
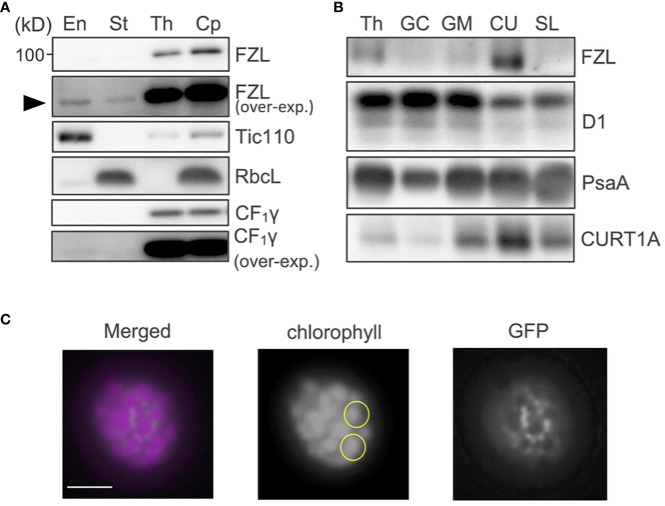
Sub-chloroplast localization of FZL. **(A)** Subfractionation of chloroplasts and immunodetection of FZL. Isolated WT chloroplasts (Cp) were separated into envelope (En), stroma (St), and thylakoids (Th). Tic110 (envelope), RbcL (stroma), and CF_1_γ (thylakoids) were detected as markers of chloroplast fractionation. Overexposed data are also shown for detection of FZL and CF_1_γ. The black arrowhead indicates the FZL signals detected in envelope and stroma fraction in the overexposed immunoblotting. **(B)** Subfractionation of thylakoids and immunodetection of FZL. Isolated WT thylakoids (Th) were treated with digitonin and separated into grana core (GC), grana margins (GM), curvature fraction (CU) and stroma lamellae (SL). D1 (grana), PsaA (stroma lamellae) and CURT1A (curvature fraction) were detected as markers of thylakoid fractionation. Loading was normalized by equal chlorophyll amount. **(C)** Confocal micrographs of a thylakoid isolated from plants expressing FZL-GFP. Each of the yellow circles marks individual granum. Bar = 1 μm.

The sub-chloroplast localization of FZL in vascular plants has been controversial as stated above, likely because it has been analyzed using an *Arabidopsis* line expressing FZL-GFP or a different plant species (pea) (Gao et al., 2006; Liang et al., 2018; Patil et al., 2018). To probe the localization of the endogenous FZL protein in *Arabidopsis*, we prepared polyclonal antibodies against FZL. Chloroplasts isolated from wild type (WT) *Arabidopsis* were fractionated into envelope, stroma and thylakoids as described in Methods, and these samples were subjected to western blotting. The results showed that FZL was predominantly localized to thylakoids (**Figure 1A**). Although it was also detected in envelope and stroma, somewhat consistent with Gao et al. (2006), the signals were very weak and only discernable in the over-exposed data. To further clarify the localization of FZL on thylakoid membranes, isolated thylakoids were treated with digitonin and separated into fractions that were pelleted by 10,000 g (grana core), 40,000 g (grana margins) and 144,000 g (a loose pellet: curvature fraction, a tight pellet: stroma lamellae), as described in Trotta et al. (2019). Western blotting of these fractions showed that FZL was enriched on curved edges of grana, in agreement with Liang et al. (2018) (**Figure 1B**). To further confirm the localization of FZL, a transgenic line expressing FZL-GFP was employed for confocal microscopic observation. The GFP signals exhibited a punctate pattern on isolated thylakoids, consistent with Gao et al. (2006), and at a higher resolution, FZL-GFP was distributed around grana, confirming the localization on grana peripheries (**Figure 1C**).

### Grana stacking is staggered and grana are less interconnected via stroma lamellae in *fzl*

**Figure 2:**
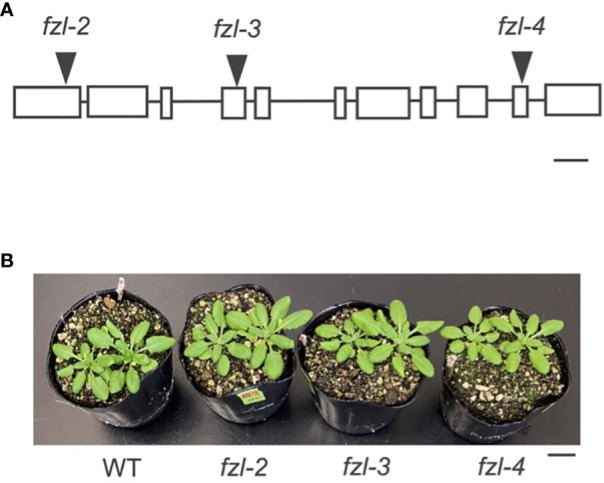
Different *fzl* mutant alleles used in this study. **(A)** A diagram showing the T-DNA insertion site in each allele. Exons are indicated by white rectangles and introns by black lines. Bar = 250 bp. **(B)** A photograph of plants. Bar = 10 mm.

**Figure 3:**
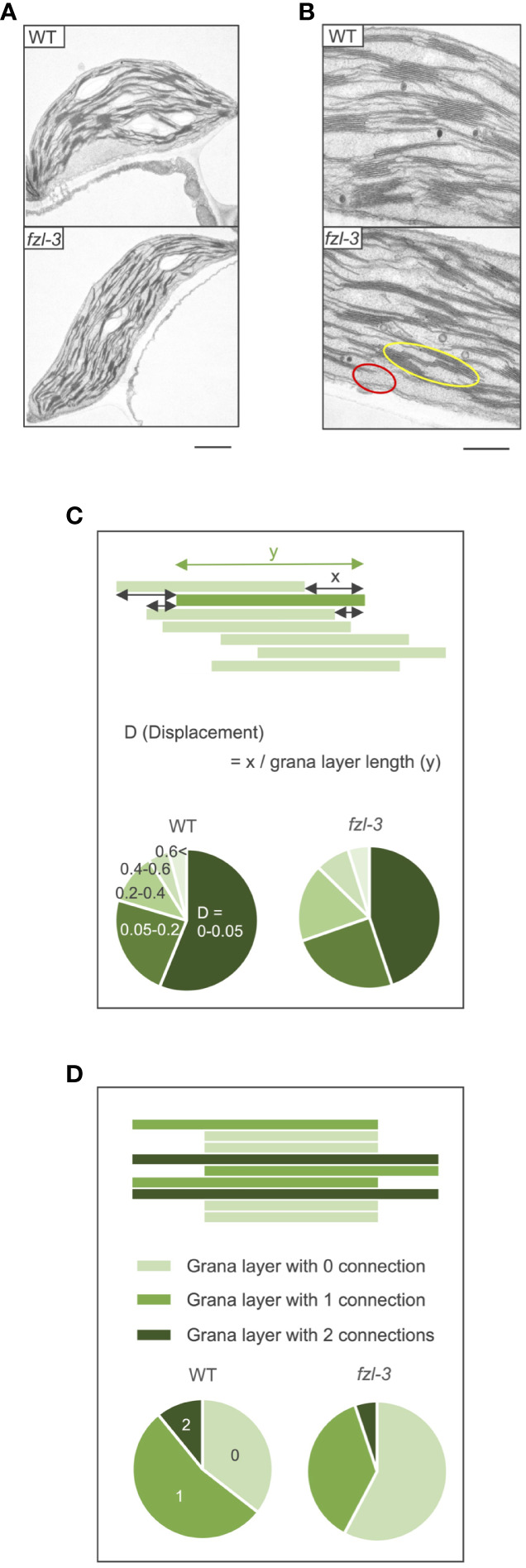
Ultrastructure of WT and *fzl-3* thylakoids. **(A)** Transmission electron microphotographs of WT and *fzl-3* chloroplasts. Bar = 1 μm. **(B)** Transmission electron microphotographs of WT and *fzl-3* thylakoids. The yellow circle marks the grana with staggered stacking and the red circle indicates the grana periphery for which interconnected stroma lamellae are not apparent on this section. Bar = 500 nm. **(C)** Comparison of staggering of grana stacking. It was quantitatively compared by measuring D (Displacement), defined as displacement lengths between grana layers indicated by the green rectangles (x) normalized against the grana layer lengths (y) (top). Distributions of D values are presented as pie charts. Darker greens represent lower D values and smaller displacements between grana layers (below) (*n* = 1051 to 1765). **(D)** Comparison of abundance of interconnections between grana and stroma lamellae. It was evaluated by frequencies of grana layers with different numbers of connections to neighboring stroma lamellae. In sectional views of grana, each layer has 0 (isolated from stroma lamellae; light green), 1 (connected to stroma lamellae at one end; green) or 2 (connected to stroma lamellae at both ends; dark green) connections (top). Distributions of grana layers with different connection numbers are presented as pie charts (below) (*n* = 310 to 515).

To characterize the function of FZL, we investigated the phenotypes of *FZL* T-DNA insertional mutants, *fzl-2* (SALK_118335C), *fzl-3* (SALK_152584C), and *fzl-4* (SALK_009051) obtained from the Arabidopsis Biological Resource Center (**Figure 2**). The leaves were identically slightly paler in these *fzl* mutants, as described in previous reports (Gao et al., 2006; Landoni et al., 2013; Tremblay et al., 2016; Patil et al., 2018), but in our plant growth conditions (90 μmol photons m^-2^ s^-1^, 8-h light/16-h dark cycles at 22°C), we did not observe the delayed growth reported in Gao et al. (2006) or the chlorophyll-deficient area confined to the midvein regions of leaves described in Patil et al. (2018) (**Figure 2B**). Given that all the *fzl* mutants also exhibited the identical photosynthetic phenotypes as mentioned below, we mainly used *fzl-3* in this study. We first reexamined the ultrastructure of mature thylakoids in WT and *fzl-3* using transmission electron microscopy (**Figure 3**). The thylakoid morphology in *fzl-3* was somewhat disorganized compared with that in WT, as reported in Gao et al. (2006), and was characterized by staggered grana stacking and less interconnections between grana and stroma lamellae (**Figures 3A, B**); the highly staggered grana (**Figure 3B**, yellow circle) and the grana peripheries devoid of apparent interconnecting stroma lamellae (**Figure 3B**, red circle) were only observed in *fzl*, but not in WT sections. These two features of *fzl* thylakoids were already implied in the previous reports Gao et al. (2006) and Liang et al. (2018), but they were just qualitatively mentioned and the latter report analyzed prothylakoids on developing stages. Therefore, we quantitatively confirmed the morphological features of the mature *fzl* thylakoids; we quantified staggering of grana stacking by measuring displacement lengths between grana layers (**Figure 3C**), and abundance of grana-stroma lamellae interlinking by counting grana layers with different numbers of connections to stroma lamellae in sectional views of grana (**Figure 3D**).

### LEF is negatively affected and NPQ is higher in *fzl*

**Figure 4:**
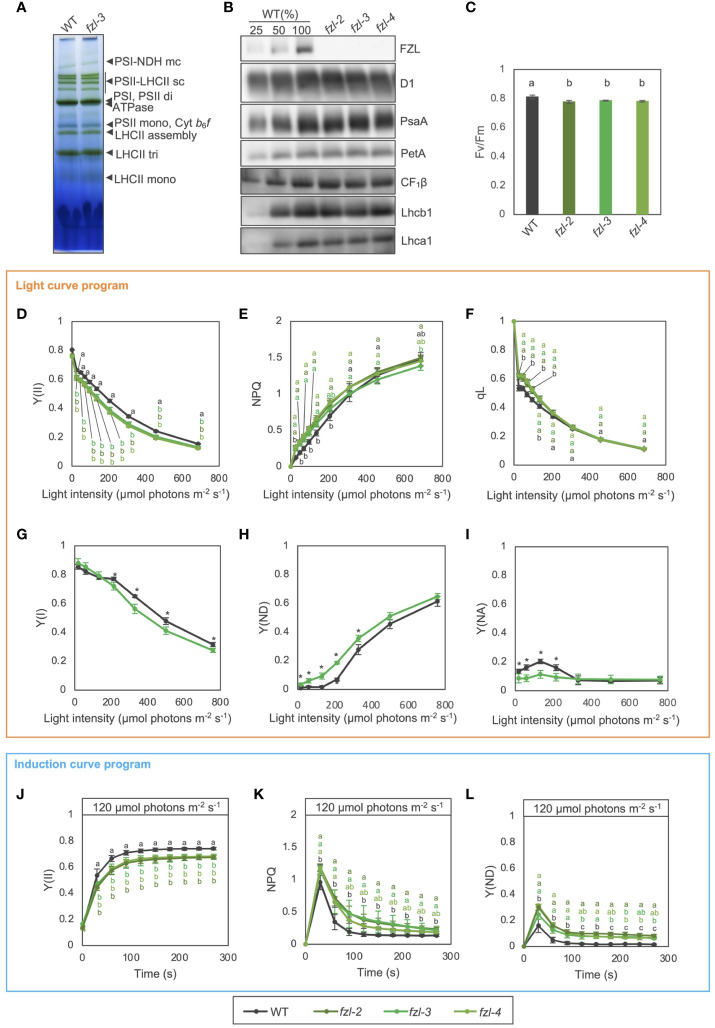
Photosynthetic phenotypes of WT and *fzl* mutants. **(A)** Separation of thylakoid protein complexes by BN-PAGE in WT and *fzl-3*. Each band was identified according to Järvi et al. (2011) and is indicated by black arrowheads at right. mc, megacomplex; sc, supercomplex; di, dimer; mono, monomer; tri, trimer. **(B)** Immunodetection of photosynthetic proteins in WT, *fzl-2*, *fzl-3* and *fzl-4*. Thylakoid proteins were analyzed, and loading was normalized by equal chlorophyll amount. For WT, dilution series of proteins were loaded. **(C)** Fv/Fm in WT and *fzl-2*, *fzl-3* and *fzl-4* mutants. Each value is the mean ± SD of 3 to 8 independent replicates. Columns with different letters are significantly different by Tukey-Kramer test (P < 0.05). **(D)** Light intensity dependence of Y(II) in WT and *fzl-2*, *fzl-3* and *fzl-4* mutants (*n* = 3 to 5). **(E)** Light intensity dependence of NPQ. **(F)** Light intensity dependence of qL. Each data point represents the mean ± SD. Different letters indicate statistical significance between genotypes at each light intensity by Tukey-Kramer test (P < 0.05). **(G)** Light intensity dependence of Y(I) in WT and *fzl-3* mutant (*n* = 3 to 5). **(H)** Light intensity dependence of Y(ND). **(I)** Light intensity dependence of Y(NA). Each data point represents the mean ± SD. Asterisks indicate statistical significance between genotypes at each light intensity by Student’s *t* test (P < 0.05). **(J)** The time course of Y(II) upon illumination at 120 μmol photons m^-2^ s^-1^ in WT and *fzl-2*, *fzl-3* and *fzl-4* mutants (*n* = 3 to 8). **(K)** The time course induction of NPQ. **(L)** The time course induction of Y(ND). Each data point represents the mean ± SD. Different letters indicate statistical significance between genotypes at each time point by Tukey-Kramer test (P < 0.05).

Accumulation of photosynthetic protein complexes was comparable between the *fzl* mutants and WT, when examined with Blue Native (BN)-PAGE and immunoblotting (**Figures 4A, B**). In chlorophyll fluorescence analysis, the maximum quantum yield of PSII (Fv/Fm) was slightly but statistically significantly lower in *fzl* (*fzl-2*; 0.776 ± 0.010, *fzl-3*; 0.785 ± 0.003, *fzl-4*; 0.780 ± 0.005) than in WT (0.810 ± 0.012) (**Figure 4C**).

To examine the *fzl* phenotypes in photosynthetic electron transfer, light intensity dependence of semi-steady state levels of chlorophyll fluorescence parameters was analyzed in WT and different *fzl* alleles using a light curve program of PAM (pulse amplitude modulation) system, in which light intensities are increased in a stepwise manner every 2 minutes (**Figures 4D–F**). In the *fzl* mutants, quantum yield of PSII (Y(II)) was reduced at all the light intensities, and NPQ was higher especially at light intensities below 370 μmol photons m^-2^ s^-1^ (**Figures 4D, E**). The qL parameter, which estimates oxidation levels of Q_A_, the primary electron acceptor of PSII (Kramer et al., 2004), was also higher in *fzl* at light intensities below 206 μmol photons m^-2^ s^-1^ (**Figure 4F**). However, qP, another indicator of Q_A_ oxidation levels (Genty et al., 1990), was similar to WT levels in *fzl* under the same light intensities, but was slightly lower in *fzl* than in WT at higher light intensities (**Supplementary Figure 1A**). The status of PSI was probed in WT and *fzl-3* by monitoring absorbance changes of PSI special chlorophyll pair (P700) using Dual-PAM (Waltz) (**Figures 4G-I**). Y(I) represents the ratio of reduced P700 which is not limited by the acceptor side, and is often used to estimate quantum yield of PSI. Y(ND) accounts for the ratio of oxidized P700. Y(NA) is the fraction of reduced P700 which cannot be oxidized by a saturation pulse, representing the acceptor side limitation of PSI. These three fractions account for the total P700, and the sum of Y(I), Y(ND) and Y(NA) is unity (Klughammer and Schreiber, 1994). In *fzl-3*, Y(ND) was higher especially at light intensities below 501 μmol photons m^-2^ s^-1^, and this was accompanied mainly by lower Y(NA) and Y(I) under lower and higher light, respectively (**Figures 4G-I**). We also investigated the time course of photosynthetic parameters during induction of photosynthesis by relatively low light (120 µmol photons m^-2^ s^-1^) using the induction curve program of dual-PAM system. We selected this light intensity because the *fzl* phenotype was clear. In agreement with the light curve data, Y(II) was lower in the *fzl* mutants than in WT (**Figure 4J**). The levels of transiently induced NPQ were higher and the relaxation was slower, and the similar trend was observed in Y(ND) (**Figures 4K, L**). The data for the other parameters were also generally consistent with the steady state data (**Supplementary Figures 1B-E**), but the high qL levels were not evident and qP was slightly lower after 120 s of illumination (**Supplementary Figures 1B, E**). In addition, Y(I) was higher in the *fzl* mutants, though this was not significant in the light curve data (**Figure 4G**, **Supplementary Figure 1C**). The suppressed Y(NA) may have led to the increase in Y(I) levels (**Supplementary Figures 1C, D**). The *fzl* photosynthetic phenotypes were complemented by expressing FZL-GFP (**Supplementary Figure 2**).

Consistent with Gao et al. (2006), chloroplast size was shown to be increased in *fzl* (**Supplementary Figure 3A**), which raised the possibility that the abnormal chloroplast size can affect photosynthetic parameters (Dutta et al., 2015; Dutta et al., 2017). To test this possibility, we performed the same analysis on other chloroplast division mutants *arc3-2* (Shimada et al., 2004) and *ftsZ1-1* (Yoder et al., 2007). The disorders in Fv/Fm values and electron transfer were not detected in these division mutants (**Supplementary Figure 3**), suggesting that the photosynthetic alterations in *fzl* are not associated with the dysregulated chloroplast divisions.

### Pmf is smaller in *fzl*, but *kea3-1* mutation enhances the *fzl* photosynthetic phenotype

**Figure 5:**
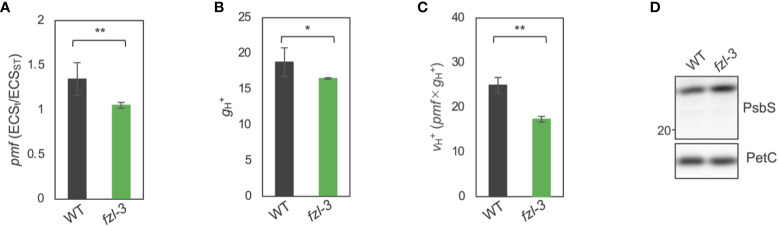
ECS analysis and immunodetection of PsbS in WT and *fzl-3* mutant. **(A)** The total size of pmf, calculated as ECS_t_/ECS_ST_. Measurements were performed after 30 s of illumination at 120 μmol photons m^-2^ s^-1^. Each value is the mean ± SD of 2 to 7 independent replicates. **(B)** Proton conductivity of thylakoid membranes ( $g_{\text{H}}^{\;+}$ ). **(C)** Proton flux of thylakoid membranes ( $v_{\text{H}}^{\;+}$ ), calculated as pmf x $g_{\text{H}}^{\;+}$ . The asterisks indicate statistical significance (*P < 0.05, **P < 0.01) by Student’s *t* test. **(D)** Immunodetection of PsbS. Thylakoid proteins were analyzed, and loading was normalized by equal chlorophyll amount. PetC was immunodetected as a loading control. For WT, dilution series of proteins were loaded.

The photosynthetic phenotypes of *fzl*, the higher NPQ and Y(ND) and the lower Y(II), led us to hypothesize that thylakoid lumen is more acidified in *fzl* than in WT, although this idea is inconsistent with the oxidation of the PQ pool suggested by the higher qL. Luminal acidification induces the qE component of NPQ and photosynthetic control monitored by Y(ND), resulting in lower Y(II) (Li et al., 2000; Munekage et al., 2001; Li et al., 2002; Yamamoto and Shikanai, 2019; Ozawa et al., 2023). Indeed, similar phenotypes have been reported in mutants with higher pmf, especially higher ΔpH. Known examples include mutants with lower accumulation levels or lower activity of chloroplast ATP synthase, in which less efficient proton efflux results in increased pmf amplitudes (Kohzuma et al., 2012; Zhang et al., 2016), and *kea3* mutants, devoid of the H^+^/K^+^ antiporter KEA3, which substitutes ΔΨ for ΔpH (Armbruster et al., 2014; Wang et al., 2017; Wang and Shikanai, 2019). To verify the hypothesis of excess luminal acidification in *fzl*, we evaluated pmf sizes in WT and *fzl-3* by measuring the decay of electrochromic shift (ECS) as described in Avenson et al. (2004). Unexpectedly, however, the light-induced pmf, calculated as ECS_t_/ECS_ST_, was lower in *fzl-3* than in WT (**Figure 5A**). The proton conductivity of thylakoid membranes, represented as $g_{\text{H}}^{\;+}$ , was smaller in *fzl-3* (**Figure 5B**). Similarly, $v_{\text{H}}^{\;+}$ , standing for the initial velocity of H^+^ flux across thylakoid membranes, was also lower in *fzl-3* (**Figure 5C**). To investigate the reason for the high NPQ in presence of the smaller pmf in *fzl*, we compared the PsbS accumulation levels in WT and *fzl* (**Figure 5D**). The result showed that they were slightly higher in *fzl*, and this may account for the enhanced NPQ. Meanwhile, Y(ND) was excessively enhanced considering the lower pmf levels in *fzl* (**Figure 4L**), suggesting additional limitations on electron transfer toward PSI other than photosynthetic control. Taken together, we consider that in *fzl*, the “additional limitations” intrinsically negatively affect LEF, contributing to the lower Y(II) and higher Y(ND) (**Figures 4D, H, J, L** **)** and that this results in the smaller pmf formation (**Figure 5A**). The delayed LEF is consistent with the smaller proton flux ( $v_{\text{H}}^{\;+}$ ) (**Figure 5C**).

**Figure 6:**
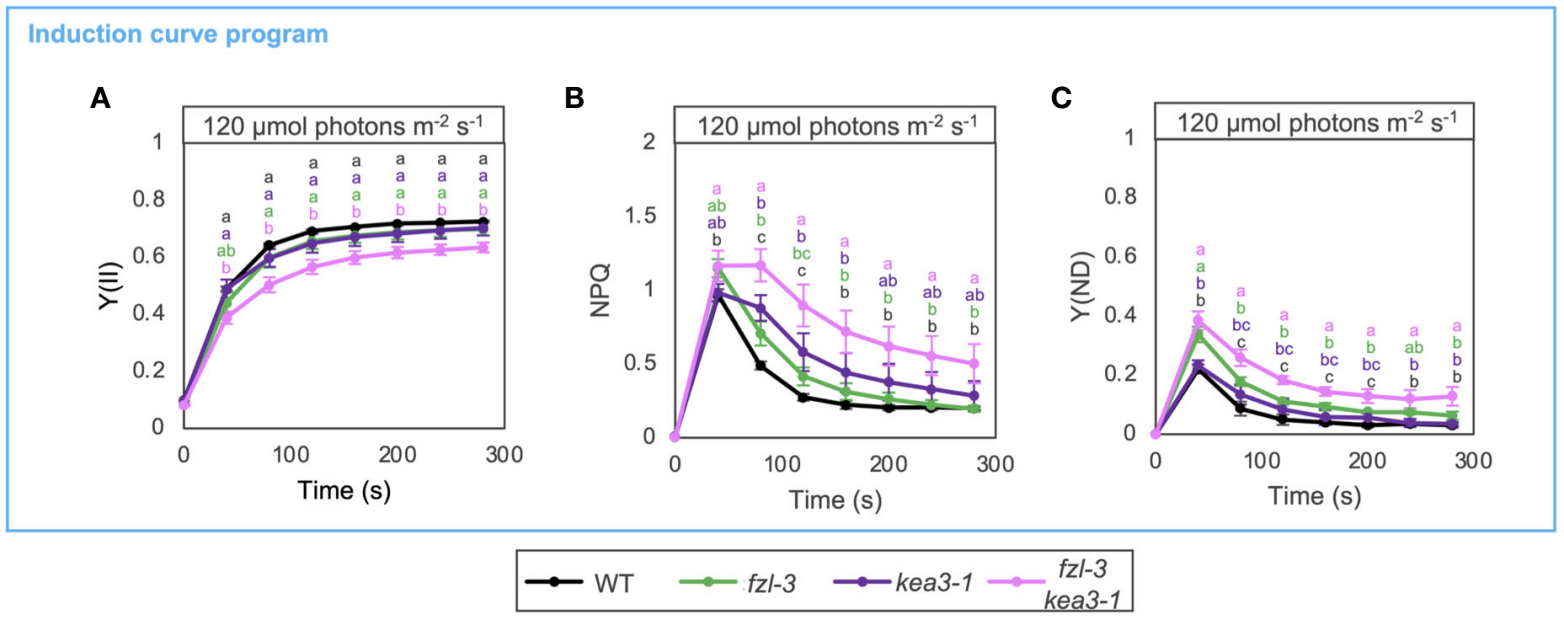
Photosynthetic phenotypes of WT, *fzl-3*, *kea3-1* and *fzl-3 kea3-1* mutants. **(A)** The time course of Y(II) upon illumination at 120 μmol photons m^-2^ s^-1^ (*n* = 3). **(B)** The time course induction of NPQ. **(C)** The time course induction of Y(ND). Each data point represents the mean ± SD. Different letters indicate statistical significance between genotypes at each time point by Tukey-Kramer test (P < 0.05).

To gain further insight into *fzl* photosynthetic phenotypes, we closely compared the kinetics of photosynthetic parameters in *fzl-3* and *kea3-1*, and also analyzed the phenotype of *fzl-3 kea3-1* double mutant (**Figure 6**). Both the single mutants indeed exhibited lower Y(II) and higher NPQ and Y(ND), though the difference was not statistically significant in Y(II) in both single mutants and Y(ND) in the *kea3-1* mutant. However, NPQ and Y(ND) were differently induced and relaxed in these mutants. In *kea3-1*, NPQ levels were higher than in WT at 40-80 s after illumination, but Y(ND) levels were similar to WT levels (**Figures 6B, C**). This is explained by the fact that a lower luminal pH is required to induce photosynthetic control than NPQ (Takizawa et al., 2007). In *fzl-3*, on the other hand, NPQ levels were almost the same as those in WT at 120-200 s after illumination, but Y(ND) levels were higher, which reflects the contribution of the additional limitations. Despite the different disorders in electron transfer in *fzl-3* and *kea3-1*, the photosynthetic phenotypes were synergistically enhanced in the *fzl-3 kea3-1*double mutant (**Figure 6**). The slightly elevated ΔpH under the *kea3-1* background may have somehow amplified the effect of the *fzl-3* defects on electron transfer, and we discuss the possible mechanisms in Discussion.

### State transitions are not affected and are not involved in the restriction on LEF in *fzl*

**Figure 7:**
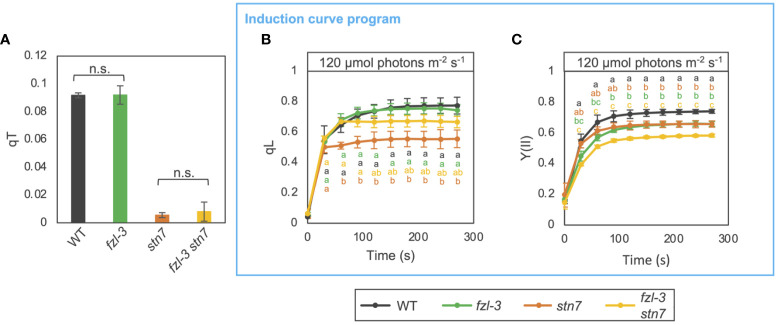
Photosynthetic phenotypes of WT, *fzl-3*, *stn7* and *fzl-3 stn7* mutants. **(A)** Quenching of chlorophyll fluorescence due to state transitions (qT). qT values were obtained as described in Pribil et al. (2010). Each value is the mean ± SD of 2 to 3 independent replicates. “n.s.” indicates no statistically significant difference by Student’s *t* test (P < 0.05). **(B)** The time course of qL upon illumination at 120 μmol photons m^-2^ s^-1^ (*n* = 2 to 5). **(C)** The time course of Y(II). Each data point represents the mean ± SD. Different letters indicate statistical significance between genotypes at each time point by Tukey-Kramer test (P < 0.05).

Given that FZL is a membrane remodeling protein localized to edges of grana margins (**Figure 1**) and that the grana-stroma lamellae interconnections were less abundant in *fzl* (**Figure 3**), lack of FZL could affect state transitions, the other short-term regulation mechanisms on electron transfer than NPQ and photosynthetic control. State transitions involves enhanced interactions between grana-enriched PSII-LHCII and stroma lamellae-enriched PSI-LHCI at grana margin regions (Tikkanen et al., 2008; Tikkanen and Aro, 2012; Grieco et al., 2015). In addition, the LHCII phosphorylation by STN7, which triggers state transitions, is known to induce another level of regulation on photosynthetic electron transfer by decreasing grana diameters to likely facilitate PQ/PC-mediated electron transfer (Wood et al., 2018; Hepworth et al., 2021). It is also possible that FZL is involved in the membrane remodeling processes. Indeed, the previous structural analysis of isolated thylakoids has suggested that the reorganization of thylakoid morphology accompanied by state transitions involves membrane fission and fusion, and the same report mentioned FZL as one of the putative players in the process (Chuartzman et al., 2008). Therefore, we evaluated the chlorophyll fluorescence change associated with state transitions evaluated by qT values, as described in Pribil et al. (2010) (**Figure 7A**). Leaves were exposed to low red light (30 μmol photons m^-2^ s^-1^) and far-red light to stimulate state 2 and state 1, respectively. Maximum fluorescence was measured in each state (Fm2 and Fm1), and qT was calculated as (Fm1-Fm2)/Fm1. qT values were similar in WT and *fzl-3*, while they were close to zero in the *stn7* mutant background, devoid of LHCII phosphorylation and state transitions (Bellafiore et al., 2005). This suggests state transitions normally occur in *fzl* despite the less grana-stroma lamellae interlinking (**Figure 3**). We also performed photosynthetic analysis using the induction curve program in WT, *fzl-3*, *stn7* and *fzl-3 stn7* (**Figures 7B, C**). Under the relatively low light conditions (120 μmol photons m^-2^ s^-1^), qL and Y(II) were lower in *stn7* than in WT, suggesting over-reduction of PQ pool and acceptor-side limitation of PSII, in accordance with previous reports (Tikkanen et al., 2010; Hepworth et al., 2021). In the *fzl-3 stn7* double mutant, the level of qL was partially restored (**Figure 7B**). In the *fzl* single mutant, the higher oxidative levels of Q_A_ under low light was observed only in the light curve data and not evident during the induction of photosynthesis, but this effect seemed to mask the over-reduction of PQ caused by lack of state transitions and by retarded electron transfer due to the larger grana (Hepworth et al., 2021). However, the *fzl* and *stn7* mutations additively lowered Y(II), suggesting that LEF is restricted in *fzl* mutant in an STN7-independent manner (**Figure 7C**).

## Discussion

### FZL likely mediates fusion of grana and stroma lamellae at grana edges

**Figure 8:**
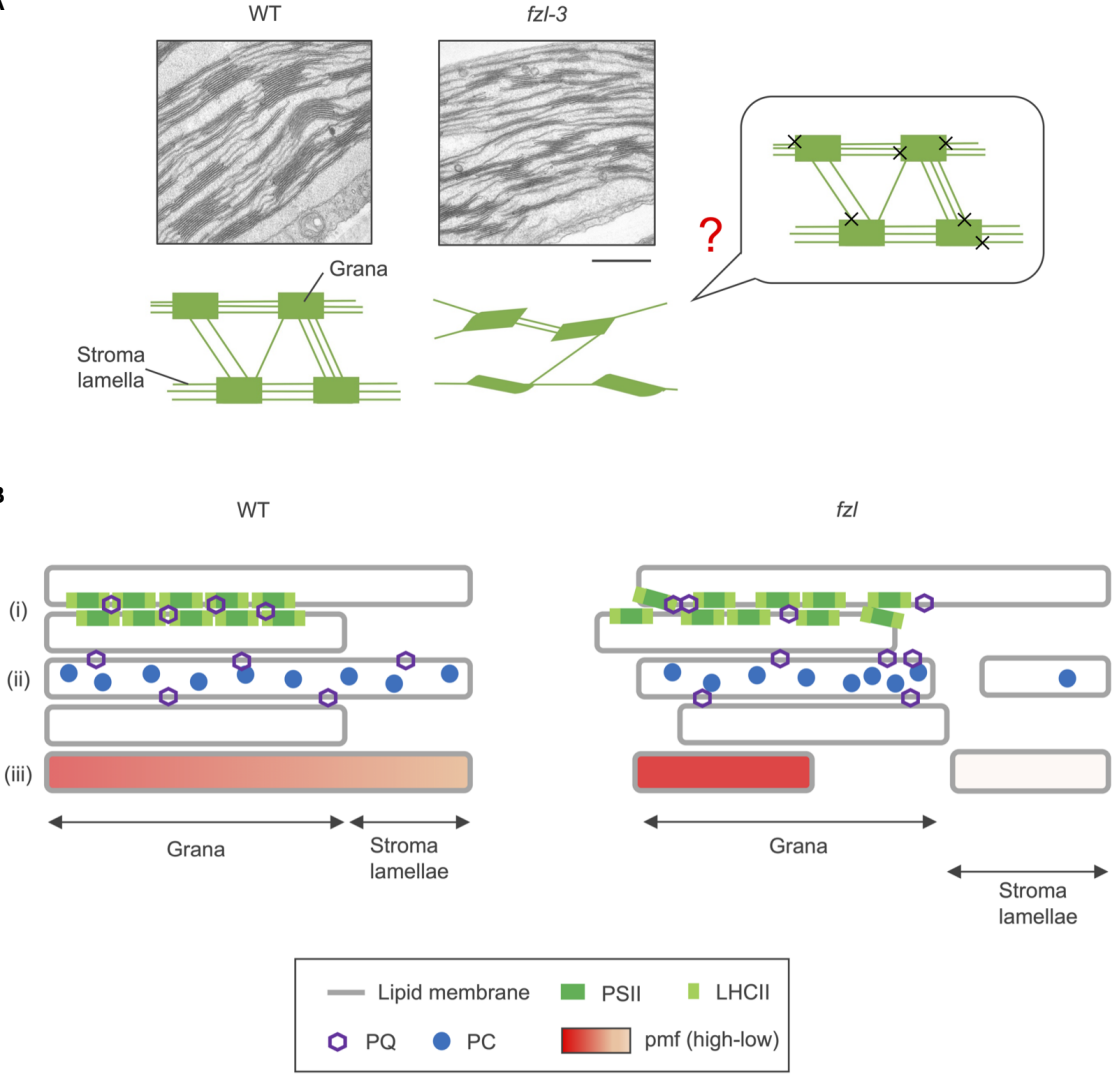
Model schemes of WT and *fzl* thylakoids. **(A)** Transmission electron microphotographs of thylakoids (top) and schematic descriptions of thylakoid morphologies (below) in WT and *fzl-3*. In the schematic picture, orderly stacked grana, staggered grana, and stroma lamellae are symbolized as rectangles, parallelograms, and lines, respectively. The less interconnections between grana via stroma lamellae are depicted in *fzl* than in WT. The putative model of how the *fzl* thylakoid morphology arises is shown on the right. The “X”s represent the absence of the fusions between grana and stroma lamellae likely mediated by FZL. The question mark indicates that the model is hypothetical and not demonstrated. Bar = 500 nm. **(B)** Schematic representation of the possible alterations in photosynthetic processes in *fzl* thylakoids (right) in comparison with those in WT thylakoids (left). In *fzl* thylakoids, (i) the staggered grana stacking might affect densely packed PSII-LHCII arrays, resulting in disturbed PQ diffusion over grana, (ii) the less frequent interconnections between grana and stroma lamellae might delay lateral diffusion of PQ and PC, and/or (iii) the fewer grana-stroma lamellae junctions could dam pmf flow from grana to stroma lamellae.

FZL, a dynamin-like protein, is likely a membrane-fusing factor in chloroplasts. FUZZY ONION/FZO, the animal and fungal homologue of FZL, is known to mediate mitochondrial fusion (Hales and Fuller, 1997; Gao et al., 2006), and the requirement of FZL in thylakoid fusion has been demonstrated in *Chlamydomonas* chloroplasts (Findinier et al., 2019). Here, we reevaluated the sub-chloroplast localization of FZL by two independent methods, subfractionation of chloroplasts and observation of GFP-fused proteins, and confirmed the enrichment on curved grana edges, which was reported in Liang et al. (2018) (**Figure 1**). The same report suggested that FZL interlinks grana and stroma lamellae at the interfaces during thylakoid biogenesis, and we presume that this model can also directly accounts for the altered morphology of the mature *fzl* thylakoids (**Figure 8A**). Lack of the grana-stroma lamellae fusions mediated by FZL (**Figure 8A**, “X”s) may lead to the less interconnections between the two membrane domains, and possibly to the staggered grana. The aligned stacking of grana sacs observed in WT could be enabled by the support from the helically surrounding stroma lamellae through the connections, and grana could be staggered without the support. However, it is difficult to demonstrate the hypothesis at present, and we leave the question mark in **Figure 8A**.

### Photosynthetic electron transfer and NPQ induction are altered in the disconnected thylakoids

Electron transfer was mildly altered in *fzl* (**Figure 4**); slower LEF was accompanied by higher NPQ and Y(ND) especially under low light. The similar tendency was previously reported in Patil et al. (2018), though they used photo-damaged samples with considerably lower Fv/Fm (0.6), making it harder to interpret other chlorophyll fluorescence parameters. The authors associated the slower LEF with the decrease in Cyt *b*_6_ *f* levels and attributed the high NPQ (they used the qN parameter instead of NPQ) to the dysregulation of carbon metabolism. However, the accumulation level of Cyt *b*_6_ *f* complex was normal in *fzl* in our data (**Figure 4B**), and the ECS analysis showed the smaller pmf formation in *fzl*, suggesting that the inefficient carbon fixation is not the primary cause of the disorder in electron transfer in *fzl* (**Figure 5A**). It has been reported that limited carbon fixation results in lower luminal pH (Avenson et al., 2004). Given the smaller pmf, the high NPQ, high Y(ND) and low Y(II) observed in the *fzl* mutants are unlikely caused by luminal acidification. As described in Results, an alternative explanation is that LEF is intrinsically retarded in *fzl* due to some kind of “additional limitations,” resulting in smaller proton budget. The concomitantly lower Y(II) and Y(I) observed at a wide range of light intensities are also consistent with delayed LEF (**Figures 4D, G**), and the significant contribution of the “additional limitations” to the high Y(ND) was clarified by comparing the kinetics of photosynthtic parameters in *fzl-3* and *kea3-1* (**Figure 6**).

What and where are the “additional limitations”? Under low light conditions, qL was higher in *fzl*, indicating that Q_A_ is more oxidized (**Figure 4F**), and this alleviated the overreduction of PQ caused by *stn7* mutation (**Figure 7B**). However, quantum yields of PSII were always lower in *fzl* (**Figure 4D**). These results suggest the defect in the reduction of Q_A_ by PSII. On the other hand, under higher light intensities, qP, another parameter reflecting oxidation levels of Q_A_ was somewhat lower in *fzl* in the light curve data (**Supplementary Figure 1A**), and the slightly lower qP was also observed 120 s after illumination with low light (120 μmol photons m^-2^ s^-1^) (**Supplementary Figure 1E**). This may suggest that acceptor-side limitations are also exerted on PSII under some conditions. Alternatively, we should possibly say that the qP phenotype is irrelevant because it is so moderate, and because qL, which is considered a better estimate of Q_A_ redox states in vascular plants (Kramer et al., 2004), was similar in WT and *fzl* when the qP differences were discernable (**Figure 4F**; **Supplementary Figures 1A, B, E**). qP is a parameter based on the ‘puddle’ model, in which each PSII reaction center possesses its own independent LHCII antenna system, while qL is derived from the lake model, where PSIIs share common LHCII antennae (Kramer et al., 2004). It is considered that the light harvesting in terrestrial plants are better approximated by the lake model, selecting qL as a more appropriate parameter. Accurately, however, the reality of light harvesting is intermediate between the two models, and the estimated Q_A_ redox levels are subject to the assumed antenna connectivity. Based on this fact, we cannot eliminate the possibility that interactions among PSII reaction centers and LHCII antennae are altered in *fzl*, making it difficult to estimate Q_A_ redox levels just by comparing qL and qP levels in WT and in *fzl*. Although we cannot determine which interpretation(s) is(are) correct about the qL/qP phenotype, taken together, there are possibilities of the defect in PSII activity, acceptor-side limitations of PSII under some conditions, and altered antenna connectivity in *fzl*. We discuss the putative mechanisms leading to these photosynthetic disorders in *fzl* as following. In *fzl* thylakoids, the stacking of grana layers is staggered (**Figures 3**, **8A**), and this might modify PSII-LHCII arrays by disturbing interactions between the complexes on opposing grana membranes (Dekker and Boekema, 2005) (**Figure 8B**i). This might lead to defects in the function of PSII, namely, the reduction of Q_A_. The functional defects may be reflected by the significantly lower Fv/Fm in *fzl* (**Figure 4C**). In addition, the alterations in the arrangement of PSII-LHCII supercomplexes could result in changes in antenna connectivity and, have been also suggested to affect the diffusion of PQ through grana (Tremmel et al., 2003) (**Figure 8B**i). Meanwhile, the less interconnections between grana and stroma lamellae in *fzl* might hamper the diffusion of PQ and PC across the domains (**Figure 8B**ii). The disturbance in the downstream electron transfer mediated by PQ/PC can result in the acceptor-side limitation of PSII.

On the other hand, NPQ was enhanced despite the smaller pmf in *fzl* (**Figures 4E, K**, **5A**), and this is likely explained by the somewhat higher PsbS levels (**Figure 5D**). The higher PsbS accumulation levels and the enhanced NPQ in presence of smaller pmf have been described in other mutants such as *ntrc* and *hcef2* (Naranjo et al., 2016; Strand et al., 2016; Nikkanen et al., 2019). Both mutants are pale green like *fzl* mutants, suggesting some defects in development and maintenance processes of chloroplasts and thylakoids. Plants might augment photoprotective strategies when chloroplast homeostasis is disturbed. Meanwhile, increasing ΔpH by crossing with *kea3-1* mutant exerted synergistic effects on the photosynthetic phenotype of *fzl* (**Figure 6**). The enhanced NPQ can be explained by the enhanced PsbS accumulation in the *fzl* background, and the considerably lower Y(II) and higher Y(ND) might be attributed to the restricted LEF due to the excess NPQ. Alternatively, the enhanced Y(ND) might reflect strengthened photosynthetic control, because the effect of NPQ to suppress LEF is generally much smaller than photosynthetic control (Tikkanen et al., 2015; Yamamoto and Shikanai, 2019). How was photosynthetic control synergistically enhanced by *kea3-1* mutation in the *fzl* mutant background, where pmf is smaller? A possible speculation is that although the whole pmf levels are lower in *fzl*, pmf could be locally higher in grana, where NPQ and part of photosynthetic control occur (**Figure 8B**iii). Grana and stroma lamellae are interconnected merely via narrow channels (so-called ‘frets’) (Bussi et al., 2019), and this has been proposed to enable localization of pmf in sub-compartments, just as pmf is locally concentrated in cristae of mitochondrial intermembrane space (Garab et al., 2022). The fewer grana-stroma lamellae junctions in *fzl* thylakoids could dam the proton flow from ‘the proton pumping domains (grana)’ with PSII to ‘the proton consuming domains (stroma lamellae)’ with ATP synthase.

Although more intensive analysis is required to determine the precise mechanisms, we showed that maintaining thylakoid connectivity and aligned grana stacking by FZL-mediated membrane fusion contributes to sustain efficient LEF and proper NPQ induction.

## Materials and methods

### Plant materials and growth conditions

*Arabidopsis thaliana* wild type (WT) plants (ecotype Columbia *gl1*), mutant plants, and transgenic plants were grown on soil for 4-5 weeks under light of 90 μmol photons m^-2^ s^-1^, 8-h light/16-h dark cycles at 22°C. The plants used for **Figure 1A** were grown on Murashige and Skoog (MS) medium containing Gelzan to facilitate isolation of intact chloroplasts. The T-DNA insertion lines *fzl-2* (SALK_118335C), *fzl-3* (SALK_152584C), *fzl-4* (SALK_009051), *arc3-2* (SALK_057144) and *ftsZ1-1*(SALK_073878) were obtained from the Arabidopsis Biological Resource Center and backcrossed three times with WT. There was disagreement in annotations of *fzl* mutant lines between Tremblay et al. (2016) and Patil et al. (2018), and therefore, we re-annotated them in accordance with the TAIR database (http://www.Arabidopsis.org/). *kea3-1*and *stn7* have been described previously in Wang et al. (2017) and Yokoyama et al. (2016), respectively. *fzl-3 kea3-1* and *fzl-3 stn7* plants were generated by crossing. The double mutants were identified in the F2 generation by PCR-based genotyping. Primers used for genotyping each gene were listed in **Supplementary Table 1**. To generate *fzl* plants expressing FZL-GFP, *FZL* cDNAs were amplified and cloned into the pENTR/D-TOPO vector (Life Technologies) using the TOPO cloning method (Life Technologies). The resulting plasmid was confirmed by sequencing and then inserted into the binary vector pFAST-R05 (Shimada et al., 2010) by LR clonase reaction (Life Technologies). *Agrobacterium tumefaciens* was transformed with the plasmids by electroporation and the bacteria were used to transform *fzl* mutant plants via the floral dip method (Clough and Bent, 1998). Transformed seeds expressing TagRFP were visually selected and T3 plants were used for analyses.

### Chloroplast and thylakoid preparation

Fresh leaves of 4- to 5-week-old *Arabidopsis* were homogenized in ice-cold grinding buffer (50 mM HEPES/KOH, pH 8.0, 330 mM sorbitol, 2 mM EDTA and 1 mM MgCl_2_). For isolation of intact chloroplasts, 5 mM Cysteine and 5 mM Ascorbate were added to grinding buffer. The homogenate was filtered through two layers of Miracloth and crude chloroplasts were precipitated by centrifugation at 2,070 g at 4°C for 5 min. To obtain thylakoid fractions, the chloroplasts were osmotically ruptured in shock buffer (50 mM HEPES/KOH, pH 8.0, 5 mM sorbitol and 5 mM MgCl_2_), followed by centrifugation at 10,000 g at 4°C for 5 min. The pellets of thylakoids were resuspended in storage buffer (50 mM HEPES/KOH, pH 8.0, 100 mM sorbitol and 10 mM MgCl_2_) and used for additional analyses. To gain total leaf extracts, leaves frozen in liquid nitrogen were pulverized with a microhomogenizer and the frozen powder was thawed in grinding buffer. The homogenate was directly used for SDS-PAGE. Chlorophyll concentration was determined as described by Porra et al. (1989).

Subfractionation of thylakoid membranes was performed according to Trotta et al. (2019) with a slight modification. Thylakoids resuspended in Tricine buffer (10 mM Tricine, pH 7.8, 10 mM NaCl and 10 mM MgCl_2_) were solubilized for 10 min at room temperature with gentle mixing with a final concentration of 0.4% [w/v] digitonin at 0.5 mg chl/mL, followed by centrifugation at 1,000 g at 4°C for 3 min to remove insoluble materials. The supernatant was subsequently centrifuged at 10,000 g at 4°C for 30 min to obtain grana core, followed by centrifugation at 40,000 g at 4°C for 30 min to collect grana margins. The supernatant fraction was further centrifuged at 144,000 g at 4°C for 90 min, resulting in a loose pellet (curvature fraction) and a tight pellet (stroma lamellae). Obtained fractions were resuspended in Tricine buffer.

For subfractionation of chloroplasts into envelope, stroma and thylakoids, the pellet of crude chloroplasts was gently resuspended in grinding buffer and loaded on top of Percoll step gradients (35 and 85% [v/v] in the same buffer also containing 0.6% [w/v] Ficoll and 1.8% [w/v] PEG4000) and spun at 2,070 g at 4°C for 20 min. Intact chloroplasts between 35 and 85% Percoll were diluted and spun for 2,070 g at 4°C for 3 min and washed once with the same buffer. The purified intact chloroplasts were lysed in hypotonic buffer (10 mM MOPS, pH 7.8 and 4 mM MgCl_2_) and the suspension was slowly loaded on top of sucrose step gradients (0.3, 0.6 and 0.93 M in the same buffer), followed by centrifugation at 70,000 g at 4°C for 1 h. Thylakoids were obtained as a pellet and soluble stromal fraction was recovered by pipetting the upper phase of gradients. Envelope membranes were collected at the 0.93/0.6 M interface and concentrated by centrifugation at 110,000 g at 4°C for 1 h after diluting three to four times in the same buffer.

### SDS-PAGE and immunoblot analysis

Protein samples were solubilized in SDS sample buffer, separated on SDS-PAGE and electro transferred onto polyvinylidene fluoride membranes. Antibodies were added, and the protein-antibody complexes were labeled using a chemiluminescence reagent (Luminata Crescendo Western HRP Substrate; Merck kGaA). The signals were detected with ChemiDoc analyzer (Bio-Rad Laboratories). The antiserum against FZL was produced by immunizing rabbits with a purified recombinant FZL (60-475 aa) protein (PhytoAB INC). Specific antibodies against CURT1A and RbcL were kindly provided by Chanhong Kim (CAS Center for Excellence in Molecular Plant Science) and Hiroshi Shimada (Hiroshima University), respectively. The antibodies against Tic110, D1 and VIPP1 were described previously (Zhang et al., 2012; Kato and Sakamoto, 2014) For detection of photosynthetic proteins PsaA, PetA, CF_1_β, CF_1_γ, PsbS, Lhcb1 and Lhca1, commercially available polyclonal antibodies were used (Agrisera).

### Microscopic observation

For observation of FZL-GFP on thylakoids, isolated thylakoids were examined using a fluorescence microscope (DSU-BX51; Olympus) equipped with a disk scanning unit. Signals from GFP and chlorophyll autofluorescence were detected with a long-path filter U-MNIBA2. The signals were separated using imageJ software. For comparison of chloroplast sizes in WT and the chloroplast division mutants, leaf tissue was fixed in 3.5% acetoaldehyde [v/v] for 1 h and macerated in 0.1M Na_2_EDTA, pH 9.0 at 60°C for 1 h to separate intact mesophyll cells. TEM analysis was conducted by the Tokai Electron Microscopy service. *Arabidopsis* rosette leaves from 4-week-old plants were cut into 2 x 2 -mm pieces and fixed in 2% [w/v] paraformaldehyde and 2% [v/v] glutaraldehyde in 0.05 M cacodylic acid buffer, pH 7.4 at 4°C overnight and postfixed in 2% [v/v] osmium tetroxide in the same buffer at 4°C for 3 h. Samples were further dehydrated with graded ethanol series (50, 70, 90 and 100%). Ethanol was subsequently replaced with a series of epoxy resin (Quetol 651; Nissin EM) dilutions (50, 70, 90 and 100%). Then, the resin was hardened for 2 d at 60°C. The chloroplast ultrastructure was evaluated on a transverse ultrathin section cut (Ultratome V; LKB Produkter). Sections were stained with 2% lead citrate and examined using a transmission electron microscope (JEM-1200EX; JEOL) at 100 kV.

### BN-PAGE

BN-PAGE was performed according to the protocol described previously with some modifications (Järvi et al., 2011). Thylakoid membranes were resuspended in 25BTH20G [25 mM BisTris/HCl, pH 7.0, 20% [w/v] glycerol, and 1.5% [w/v] n-dodecyl-β-D-maltoside (DM)] at 1 mg chl/mL. Thylakoids were solubilized for 5 min on ice. After centrifugation at 15,000 g at 4°C for 10 min, the supernatant was supplemented with one-tenth volume of sample buffer (100 mM BisTris/HCl, pH 7.0, 0.5 M 6-amino-caproic acid, 30% [w/v] sucrose and 50 mg/mL Coomassie Brilliant Blue G-250) and loaded onto a 4 to 16% gradient native gel. Electrophoresis was conducted at 4°C, and the voltage was 50V at the beginning and increased by 25V every 30 min. Anode buffer (50 mM BisTris/HCl, pH 7.0) and Cathode buffer (50 mM Tricine, 15 mM BisTris and 0.01% Coomassie Brilliant Blue G-250) were used, and 90 min after beginning, the cathode buffer was exchanged for the one without Coomassie Brilliant Blue G-250.

### *In vivo* measurements of chlorophyll fluorescence and P700 absorption changes

For analysis of the light intensity dependence of fluorescence parameters, chlorophyll fluorescence was measured using a mini-PAM II portable chlorophyll fluorescence fluorometer (Walz). Minimum fluorescence from open PSII centers (Fo) in dark-adapted states was excited by a weak measuring light (ML) (red light, 654 nm, 0.05-0.1 μmol photons m^-2^ s^-1^). A saturating pulse (SP) of red light (800 ms, 3,000 μmol photons m^-2^ s^-1^) was applied to determine maximum fluorescence from closed PSII centers in dark-adapted state (Fm) and during red actinic light (AL) illumination (Fm’). Steady-state fluorescence level (Fs) was recorded during AL illumination. The intensity of AL was increased in a stepwise manner every 2 min (26–685 μmol photons m^-2^ s^-1^) after application of a saturating pulse. Fv/Fm was calculated as (Fm – Fo)/Fm. Y(II) and NPQ were calculated as (Fm’ - Fs)/Fm’ and (Fm - Fm’)/Fm’, respectively (Genty et al., 1989). qP and qL were calculated as (Fm’-Fs)/(Fm’-Fo’) and (Fo’/Fs) x qP, respectively (Kramer et al., 2004). To probe the time-course kinetics of photosynthetic parameters after dark-to-light transition, chlorophyll fluorescence and chlorophyll P700 absorption changes in PSI were simultaneously measured using Dual-PAM 100 (Waltz). The light intensity dependency of PSI parameters was also analyzed using Dual-PAM 100 (Waltz). Plants were dark adapted more than 30 min before measurements, and detached leaves were used for analyses. Red measuring light (620 nm) and AL (635 nm, 120 μmol photons m^-2^ s^-1^) were used and an SP of red light (300 ms, 10,000 μmol photons m^-2^ s^-1^) was applied to determine Fm and Fm’. Redox change of P700 was assessed by monitoring absorbance change of transmission light at 830 and 875 nm. Pm (the level of P700 signal of maximal oxidizable P700) was determined by application of an SP in presence of far-red light (720 nm). Maximal level of oxidized P700 during AL illumination (Pm’) was determined by SP applications. Steady state P700 signal P was recorded immediately before an SP. Y(I) was calculated as (Pm’ – P)/Pm. Y(ND) was calculated as P/Pm. Y(NA) was calculated as (Pm – Pm’)/Pm. Three complementary quantum yields were defined as follows: Y(I) + Y(ND) + Y(NA) =1 (Klughammer and Schreiber, 1994).

Quenching of chlorophyll fluorescence due to state transition (qT) was determined according to Pribil et al. (2010) with a slight modification, and Dual-PAM 100 (Waltz) was used. Dark-adapted leaves were illuminated with red light (30 μmol photons m^-2^ s^-1^, 10 min) and then maximum fluorescence in state 2 (Fm2) was measured. Next, state 1 was induced by far-red light (maximal light intensity corresponding to level 20 in the Dual-PAM setting, 10 min), and Fm1 was recorded. qT was calculated as (Fm1-Fm2)/Fm1.

### ECS analysis

ECS measurements were carried out using a Dual-PAM 100 equipped with a P515/535 module (Walz). Analysis was conducted as described in Avenson et al. (2004). Plants were dark adapted more than 30 min before measurements, and detached leaves were used for analyses. ECS signals were obtained after 30 s of illumination of red AL of 120 μmol photons m^-2^ s^-1^. ECS_t_ represents the size of light-induced pmf and was estimated from the total amplitude of the rapid decay of ECS signal after AL was turned off. ECS signal levels were normalized against a 515-nm absorbance change induced by a single turnover flash (ECS_ST_), which was measured on dark-adapted leaves before recording. $g_{\text{H}}^{\;+}$ , which reflects proton conductivity of ATPase, was estimated by fitting the first 300 ms of the decay curve with a first-order exponential decay kinetic as the inverse of decay time constant. $v_{\text{H}}^{\;+}$ , representing proton flux of thylakoid membranes, was calculated as pmf x $g_{\text{H}}^{\;+}$ .

### Statistical analyses

Statistical analyses were performed using Turkey-Kramer test and Student’s *t* test.

## Data availability statement

The raw data supporting the conclusions of this article will be made available by the authors, without undue reservation.

## Author contributions

YO: Conceptualization, Data curation, Formal Analysis, Funding acquisition, Investigation, Methodology, Project administration, Validation, Visualization, Writing – original draft, Writing – review & editing. MI: Methodology, Supervision, Writing – review & editing. TS: Conceptualization, Project administration, Resources, Supervision, Validation, Writing – review & editing. WS: Conceptualization, Data curation, Funding acquisition, Project administration, Resources, Supervision, Validation, Writing – review & editing.
