# Supplementary material for: FZL, a dynamin-like protein localized to curved grana edges, is required for efficient photosynthetic electron transfer in Arabidopsis
Source: Front Plant Sci. 2023 Sep 28;14:1279699. doi: 10.3389/fpls.2023.1279699 (PMC10568140; doi:10.3389/fpls.2023.1279699)
Supplement: Supplementary file 1 [file DataSheet_1.zip › FZL frontiers sup/Supplementary_TABLE_1.pdf]

Primer names	Sequence 5' to 3'
SALK-LBb1	GCGTGGACCGCTTGCTGCAACT
SALK-LBb1.3	ATTTTGCCGATTTCGGAAC
GABI-LB	ATATTGACCATCATACTCATTGC
fzl-2-LP	CCGCCGCATCTCCACCGTTTCCTGGCCG
fzl-2-RP	CAAGACTCTGGCGAAGAGCTACATCCC
fzl-3-LP	GGAGGAAGTCTCCCTTCTAATTGATGC
fzl-3-RP	GAAGGATCACATTGGTCCCAGGTGTG
fzl-4-LP	GCTGAATGGCTACAATCAAATACTGCCC
fzl-4-RP	GCTGAGCTTCTTCTCGATAAGGCTTGG
kea3-1-LP	TTGTCCCTGCATTCAGAATTC
kea3-1-RP	GCCAAATCTTGTAGGGAGCTC
stn7-LP	GAGCTTGTGGGAATAGCTGTG
stn7-RP	TAGTTGAACATGCGTGAGTCG
FZL-comple-FW	CACCATGAGAACTCTAATCTCTCAC
FZL-comple-REV	AAGTCTCATCTCGTCTCGTG

Supplementary Table 1. List of primers used in this study.
