# Supplementary material for: FZL, a dynamin-like protein localized to curved grana edges, is required for efficient photosynthetic electron transfer in Arabidopsis
Source: Front Plant Sci. 2023 Sep 28;14:1279699. doi: 10.3389/fpls.2023.1279699 (PMC10568140; doi:10.3389/fpls.2023.1279699)
Supplement: Supplementary file 1 [file DataSheet_1.zip › FZL frontiers sup/Supplementary_FIGURE_3.pdf]

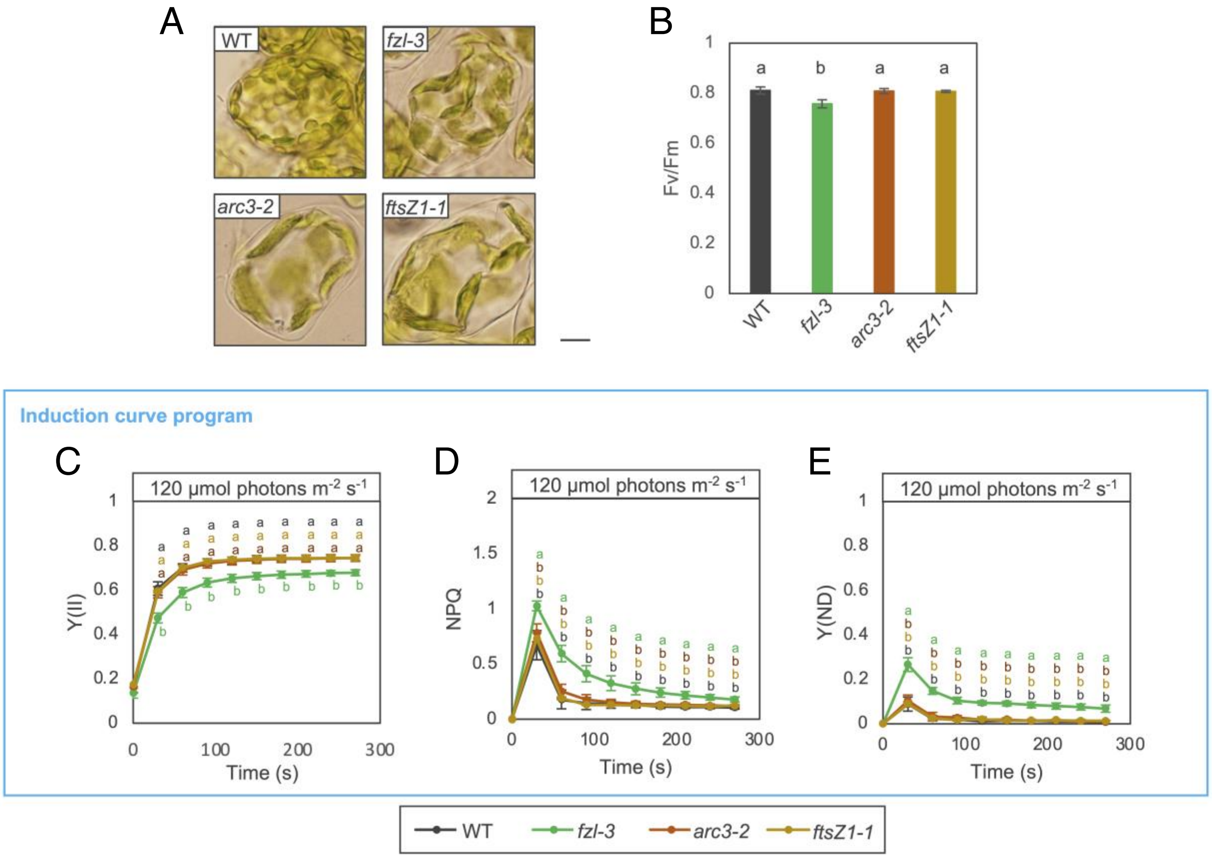

Supplementary Figure 3. Photosynthetic phenotypes of WT and *fzl-3*, *arc3-2* and *ftsZ1-1* mutants. (A) Differential interference micrographs of isolated mesophyll cells. Bar = 10 µm. (B) Fv/Fm. Each value is the mean ± SD of 3 to 7 independent replicates. Columns with different letters are significantly different by Tukey-Kramer test (P < 0.05). (C) The time course of Y(II) upon illumination at 120 µmol photons m^{-2} s^{-1} (n =3 to 7). (D) The time course induction of NPQ. (E) The time course induction of Y(ND). Each data point represents the mean ± SD. Different letters indicate statistical significance between genotypes at each time point by Tukey-Kramer test (P < 0.05).
