# Supplementary material for: FZL, a dynamin-like protein localized to curved grana edges, is required for efficient photosynthetic electron transfer in Arabidopsis
Source: Front Plant Sci. 2023 Sep 28;14:1279699. doi: 10.3389/fpls.2023.1279699 (PMC10568140; doi:10.3389/fpls.2023.1279699)
Supplement: Supplementary file 1 [file DataSheet_1.zip › FZL frontiers sup/Supplementary_FIGURE_1.pdf]

frontiers

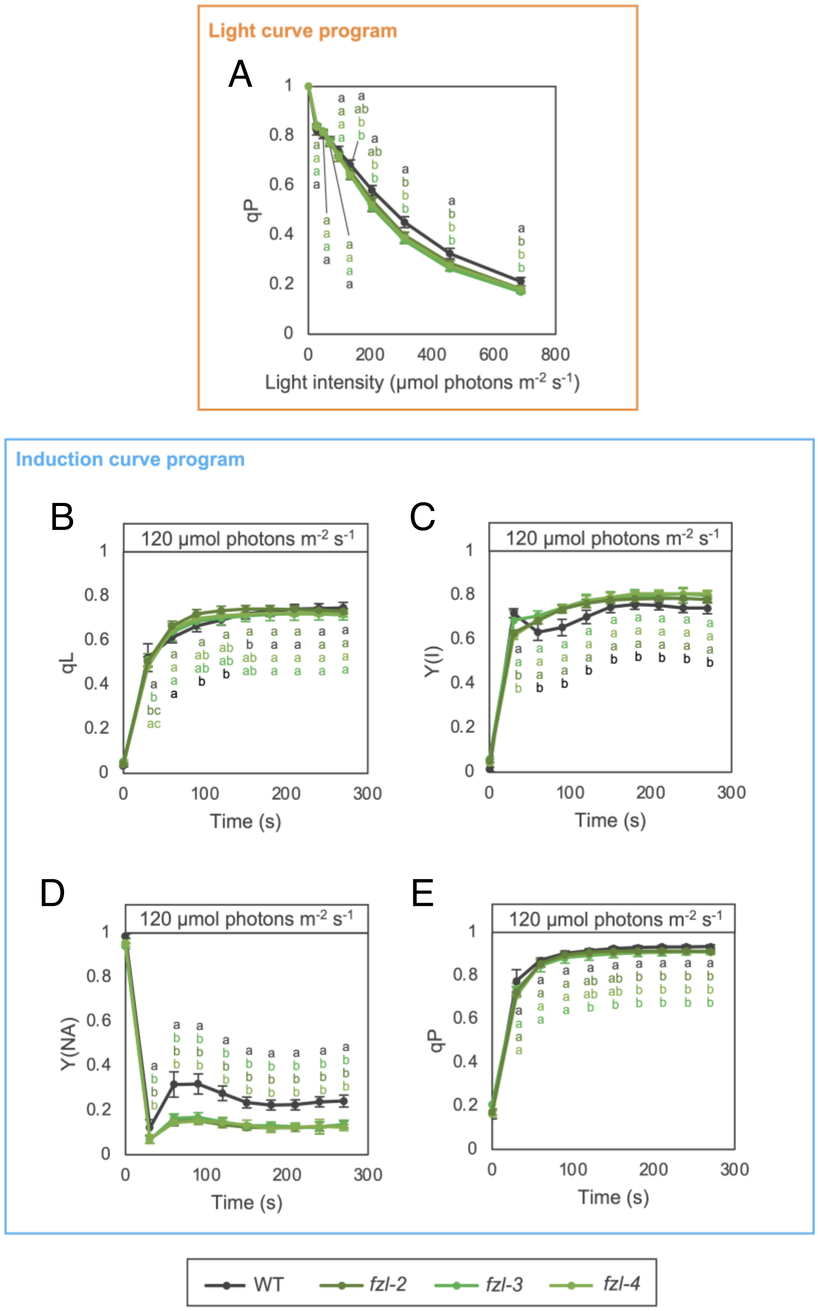

Supplementary Figure 1. Additional data for photosynthetic phenotypes of WT and *fzl* mutants. (A) Light intensity dependence of qP in WT and *fzl-2*, *fzl-3* and *fzl-4* mutants (n = 3 to 6). Each data point represents the mean ± SD. Different letters indicate statistical significance between genotypes at each light intensity by Tukey-Kramer test ($P < 0.05$). (B) The time course of qL upon illumination at 120 μmol photons m^{-2} s^{-1} in WT and *fzl-2*, *fzl-3* and *fzl-4* mutants (n = 3 to 8). (C) The time course of Y(I). (D) The time course of Y(NA). (E) The time course of qP. Each data point represents the mean ± SD. Different letters indicate statistical significance between genotypes at each time point by Tukey-Kramer test ($P < 0.05$).
