# Supplementary material for: FZL, a dynamin-like protein localized to curved grana edges, is required for efficient photosynthetic electron transfer in Arabidopsis
Source: Front Plant Sci. 2023 Sep 28;14:1279699. doi: 10.3389/fpls.2023.1279699 (PMC10568140; doi:10.3389/fpls.2023.1279699)
Supplement: Supplementary file 1 [file DataSheet_1.zip › FZL frontiers sup/Supplementary_FIGIRE_2.pdf]

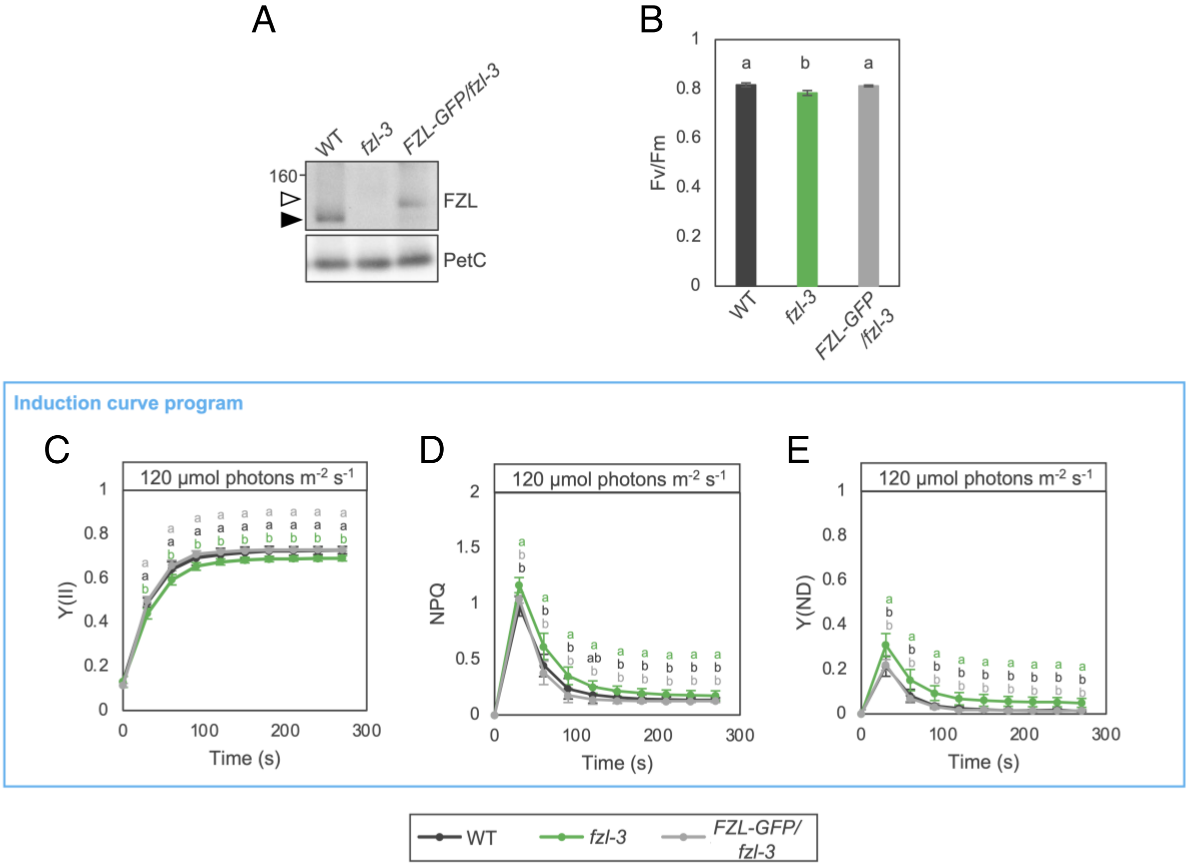

Supplementary Figure 2. Photosynthetic phenotypes of WT, *fzl-3* and *FZL-GFP*/*fzl-3*. (A) Immunodetection of FZL (closed arrowhead) and FZL-GFP (open arrowhead) using anti-FZL antibody. PetC was detected as a loading control. Total leaf extracts were analyzed, and loading was normalized by equal chlorophyll amount. (B) Fv/Fm. Each value is the mean ± SD of 7 to 10 independent replicates. Columns with different letters are significantly different by Tukey-Kramer test ($P < 0.05$). (C) The time course of Y(II) upon illumination at 120 µmol photons m^{-2} s^{-1} (n = 7 to 10). (D) The time course induction of NPQ. (E) The time course induction of Y(ND). Each data point represents the mean ± SD. Different letters indicate statistical significance between genotypes at each time point by Tukey-Kramer test ($P < 0.05$).
